# Nitric Oxide Level Is Self-Regulating and Also Regulates Its ROS Partners

**DOI:** 10.3389/fpls.2016.00316

**Published:** 2016-03-17

**Authors:** María C. Romero-Puertas, Luisa M. Sandalio

**Affiliations:** Department of Biochemistry and Cellular and Molecular Biology of Plants, Estación Experimental del Zaidín (Consejo Superior de Investigaciones Científicas)Granada, Spain

**Keywords:** nitration, nitric oxide, peroxynitrite, plant, post-translational modifications, reactive oxygen species, *S*-nitrosylation, stress

## Introduction

Nitric oxide (NO) is a free radical recognized as an omnipresent inter- and intra-cellular signaling molecule involved in the regulation of an extraordinary range of diverse cellular functions in plants (Besson-Bard et al., 2008). All NO derivatives are called reactive nitrogen species (RNS) which include more favorable structures such as nitrosonium cation (NO^+^) and nitroxyl radical (NO^−^) as a result of NO gaining or losing electrons, and the products of the reaction between NO and its close partners, reactive oxygen species (ROS), such as peroxynitrite (ONOO^−^), and the NO_x_ compounds (NO_2_, N_2_O_3_, and N_2_O_4_; Bellin et al., 2013). Although it is known that NO may regulate gene transcription and activate secondary messengers, the ways in which NO functions are still mostly unidentified (Besson-Bard et al., 2008; Palmieri et al., 2008; Gaupels et al., 2011). In the last decade, however, it has been shown that NO is also able to control different biological processes in plants by directly modifying proteins through covalent post-translational modifications (PTMs) giving rise to nitration, nitrosylation or *S*-nitrosylation (Romero-Puertas et al., 2013). *S*-nitrosylation is the covalent binding of a NO group to a cysteine residue and probably the better known NO-dependent PTM as more than 1000 proteins have been shown as targets of *S*-nitrosylation (Kovacs and Lindermayr, 2013), although the functional effect of this modification has only been analyzed in around 2% of these proteins (Astier et al., 2012; Kovacs and Lindermayr, 2013; Romero-Puertas et al., 2013). Nitration, in which 3-nitrotyrosine is produced after a nitrite group is added to the ortho-position of Tyr residues, is another NO-dependent PTM, although so far analyzed to a lesser degree than *S*-nitrosylation (N-Tyr; Vandelle and Delledonne, 2011). These PTMs are able to modify the activity, location, aggregation, or even stability of proteins (de Pinto et al., 2013; Gibbs et al., 2014; Albertos et al., 2015). We should bear in mind that NO function depends on the rate and location of its production and that NO level will determine a cytotoxic or stimulating effect (Beligni and Lamattina, 2001; Serrano et al., 2012). Thus, a precise control of NO level by switching the NO signaling off or not seems to be a crucial event for plant survival, and it appears that plants have developed many strategies to achieve it.

Reactive oxygen species (ROS) comprises oxygen derivatives species produced by the reduction of oxygen, such as superoxide radicals (O${}_{2}^{.-}$), hydroxyl radicals (·OH), peroxyl radicals (ROO·), and alkoxyl radicals (RO·) and also some non-radical compounds such as hydrogen peroxide (H_2_O_2_), the singlet oxygen (^1^O_2_), ozone (O_3_), and hypochlorous acid (HOCl^−^) (Halliwell and Gutteridge, 2007). Although research on ROS, which have strong oxidizing potential, initially focused on cytotoxicity, in recent years, it has become clear that they can also function as signaling molecules in most cellular processes (Baxter et al., 2014). Thus, plants have developed a means of utilizing lower concentrations of ROS as signaling molecules under certain physiological and stress conditions (Petrov and Van Breusegem, 2012). Genetic and pharmacological techniques have demonstrated that different ROS species can affect nuclear gene expression by responding to a variety of environmental stimuli (Sandalio and Romero-Puertas, 2015). However, a finely tuned balance between ROS scavenging and ROS production is necessary to determine their level and impact as damaging and signaling molecules (de Pinto et al., 2012; Baxter et al., 2014).

## Nitric oxide levels are self-regulating

**Figure 1:**
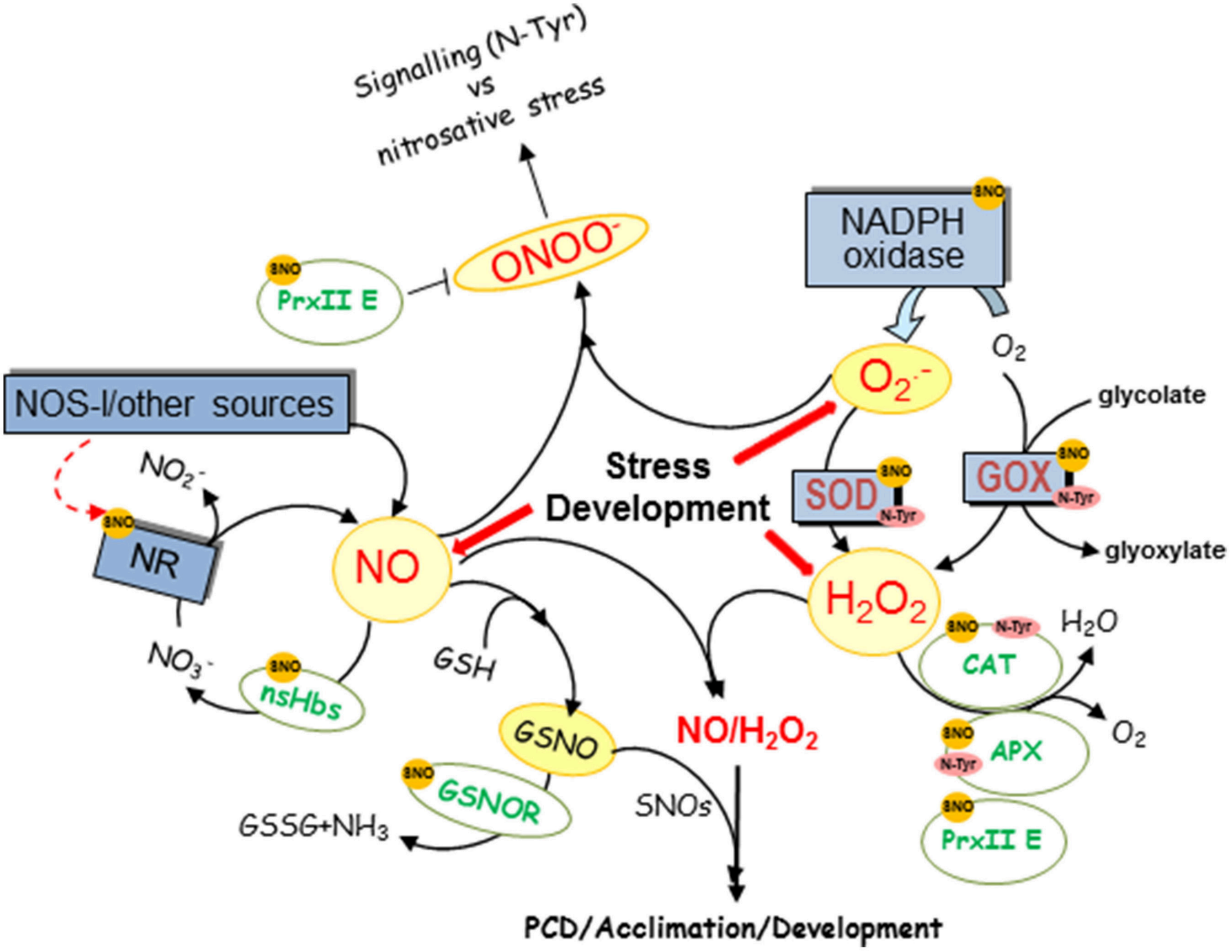
**Overview of NO and ROS level regulation by NO**. NO regulates through posttranslational modifications (PTMs), NO and ROS producing and scavenging enzymes and the figure shows a diagram of the main targets of S-nitrosylation (SNO), nitrosylation (Haem-NO), or nitration (N-Tyr) described in plants. CAT, catalase; GSH, glutathione; GOX, glycolate oxidase; GSNO, nitrosoglutathione, GSNOR, GSNO reductase; nsHbs, non-symbiotic hemoglobins; NOS-l, activity that resembles NO production as catalyzed by the animal enzyme NOS; NiNOR, plasma membrane-bound NiNOR; NR, nitrate reductase; XOR, xanthine oxidoreductase.

NO is generated in higher plants through a variety of mechanisms and both, oxidative (arginine or hydroxylamine-dependent) and reductive (nitrate-dependent) pathways have been described (Fröhlich and Durner, 2011; Gupta et al., 2011a) nitrate reductase (NR) being the best known pathway for NO production in plants (Rockel et al., 2002). NR is capable of reducing nitrite to NO depending on nitrite accumulation and pH levels. Moreover, NO can react reversibly with glutahione (GSH) producing GSNO, a reservoir of NO (Liu et al., 2001; Sakamoto et al., 2002). GSNO is metabolized by GSNO reductase (GSNOR) which controls NO and nitrosothiol levels, being a key enzyme in most NO-regulated processes, such as pathogen defense, root development, and nitrogen assimilation (Feechan et al., 2005; Rustérucci et al., 2007; Frungillo et al., 2014). It has recently been shown that GSNO inhibits nitrate uptake and its reduction to nitrite which would prevent NR-dependent NO production (Figure 1; Frungillo et al., 2014). Additionally, CO_2_ elevation distinctly increased *S*-nitrosylated NR levels in plants grown under high-nitrate conditions, along with a significant decrease in NR activity similarly to that which ocurrs with chilling treatment (Cheng et al., 2015; Du et al., 2015). These results suggest that *S*-nitrosylation of NR may decrease NR activity. Interestingly, NR regulation in response to high CO_2_ levels is nitric oxyde synthase-like (NOS_l_)-dependent (Du et al., 2015) pointing to a regulation between the different NO-production pathways. Moreover, NO also could activate NR activity under a relative low-nitrate concentration through the interaction with the haem and molybdenum centers in NR, which enhances electron transfer during nitrate reduction (Du et al., 2008). To complete the cycle, it has been also demonstrated that NO inhibits GSNOR1 through *S*-nitrosylation avoiding at the same time GSNO degradation and regulating plant nitrosothiol levels (Figure 1; Frungillo et al., 2014). Thus, (S)NO feedback regulates nitrogen flux through nitrite assimilation pathways and controls its bioavailability by modulating its own consumption (Figure 1; Frungillo et al., 2014). In the context of hypersensitive response, NO is also able to regulate the level of its own radicals, such as ONOO^−^ through *S*-nitrosylation of Arabidopsis peroxiredoxin II E (PrxII E) that inhibits its H_2_O_2_-reducing and peroxinitrite-detoxifying activities (Romero-Puertas et al., 2007).

In addition, non-symbiotic haemoglobins (nsHbs) from different species have been shown to metabolize NO, which are able to move to a solution producing nitrate (Perazzolli et al., 2006; Gupta et al., 2011b). The expression of nsHb1 is induced under low oxygen stress in different plant species when an increase in NO due to NR is assumed to occur (Gupta et al., 2011a) and *Arabidopsis thaliana* nonsymbiotic hemoglobin (AHb1) scavenges NO through production of S-nitrosohemoglobin under hypoxic stress (Perazzolli et al., 2004).

## Nitric oxide regulates reactive oxygen species

Besides its signaling functions ROS can act as oxidizing agents on proteins, lipids and nucleic acids modifying the activity or function of these molecules and hence, the steady-state levels of ROS must be strongly regulated by scavenging systems including enzymatic and non-enzymatic antioxidants, such as superoxide dismutases (SOD) that are involved in removing superoxide radicals, catalase (CAT), and the ascorbate-glutathione cycle (ASC-GSH) made up of ascorbate peroxidase (APX), monodehydroascorbate reductase (MDHAR), dehydroascorbate reductase (DHAR), glutathione reductase (GR), ascorbate (ASC), and glutathione (GSH; Jimenez et al., 1997; Romero-Puertas et al., 2006) involved in maintaining H_2_O_2_ levels under control.

NO has a half-life of only a few seconds and, once produced, interacts rapidly with ROS, giving rise to a number of RNS, such as nitrogen dioxide (NO_2_), which degrade to nitrite (a precursor to NO) and nitrate in aqueous solutions (Figure 1; Neill et al., 2008), ONOO^−^ after reaction with O${}_{2}^{.-}$ radical and other NO_x_ species. Thus, the function of NO is very much linked to ROS and the first evidence of NO and ROS crosstalk was shown during hypersensitive response (HR) in which NO and H_2_O_2_ cooperates to trigger hypersensitive cell death and an appropriate balance between ROS and NO production is required (Delledonne et al., 2001). Actually, during the HR, SOD accelerates O${}_{2}^{.-}$ dismutation to H_2_O_2_ to minimize the loss of NO by reaction with O${}_{2}^{.-}$ and to trigger hypersensitive cell death. Surprisingly, very recently it has been shown that when S-nitrosothiols are high during the HR, nitric oxide governs a negative feedback loop limiting the hypersensitive response by *S*-nitrosylation of the NADPH oxidase, the enzyme that produces O${}_{2}^{.-}$ radicals (Figure 1; Yun et al., 2011). NO is not only able to regulate O${}_{2}^{.-}$ production but also H_2_O_2_ as it has been shown that the *S*-nitrosylation pattern of glycolate oxidase (GOX), one of the main H_2_O_2_ sources in the peroxisome, changes in response to Cd (Ortega-Galisteo et al., 2012).

A number of antioxidant enzymes have been shown to be regulated by NO, meaning that NO is also able to modulate ROS levels by regulating the antioxidant system. Thus, peroxynitrite inhibits through Tyr nitration the mitochondrial manganese SOD1 (MSD1), peroxisomal copper/zinc SOD3 (CSD3), and chloroplastic iron SOD3 (Holzmeister et al., 2015). Although SODs have been identified as candidates for *S*-nitrosylation in different species, the effect of this PTM on the function of SODs has not so far been confirmed (Lindermayr et al., 2005; Tanou et al., 2009; Sehrawat et al., 2013), and the activity appears to be unaffected by GSNO in Arabidopsis recombinant proteins (Holzmeister et al., 2015). Additionally, catalase (CAT), one of the main enzymes involved in degrading H_2_O_2_ produced in peroxisomes, is *S*-nitrosylated and nitrated, causing both PTMs a loss of protein activity (Lozano-Juste et al., 2011; Ortega-Galisteo et al., 2012; Chaki et al., 2015). NO can react with the haem group of proteins, being CAT and APX reversibly inhibited during the resistance response, thus supporting a role for NO in regulating H_2_O_2_ levels in this context (Clark et al., 2000). All the enzymes of the ASC-GSH cycle also appear to be regulated by NO through *S*-nitrosylation and/or nitration, APX being the one directly involved in H_2_O_2_ detoxification and the most studied (Correa-Aragunde et al., 2015). Whilst APX activity has been shown to be inhibited by nitration (Begara-Morales et al., 2014) differing results have been found regarding the effect of *S*-nitrosylation on APX. Thus, *S*-nitrosylation of APX avoids carbonylation of the protein in seeds of *A. toxicaria* (Bai et al., 2011), increases its activity in salt stressed pea plants (Begara-Morales et al., 2014), enhances its activity by increasing resistance to oxidative stress and playing an important role in regulating immune responses (Yang et al., 2015), and under heat shock and H_2_O_2_ conditions during PCD in tobacco BY2 cells an inhibition of enzyme activity by *S*-nitrosylation, ubiquitination and degradation of APX has been described (de Pinto et al., 2013). It has also been shown that APX1 denitrosylation causes the partial inhibition of APX1 activity during root development (Correa-Aragunde et al., 2013). From these results it appears that the level of stress conditions (Correa-Aragunde et al., 2015), specific environment or possibly the species involved may affect the effect of *S*-nitrosylation on APX.

## Conclusions

In most NO regulated processes, plant response is not activated by NO alone but is the result of a network of connections between different signaling molecules and pathways, especially the ROS-dependent ones. In many cases, the level of NO, ROS, and their balance will determine cell fate and recent research has uncovered several key NO-dependent PTMs showing that NO is able to self-regulate and also regulates ROS levels, allowing the plant to fine-tune specific responses to different stimuli. However the identification of additional components of NO regulated networks, especially those that are involved in ROS metabolism, is required to fully understand this process. Capturing the whole scene may not be an easy task however, and some questions remain to be answered. Although NO has been shown to be able to modify proteins through different PTMs, it is still unclear what triggers a specific PTM in response to a specific signal and how crosstalk between different PTMs in the same protein is regulated. NO-dependent PTMs have also been shown to affect the stability of certain proteins. However, as has been demonstrated in relation to many hormonal pathways, we still do not know whether ubiquitin-mediated protein degradation is a central regulatory mechanism in NO-signaling. Further study is required to clarify this issue.

## Author contributions

MP wrote the article and LS discussed and commented on the manuscript.

### Conflict of interest statement

The authors declare that the research was conducted in the absence of any commercial or financial relationships that could be construed as a potential conflict of interest.

## References

[B1] Albertos P. Romero-Puertas M. C. Tatematsu K. Mateos I. Sánchez-Vicente I. Nambara E.. (2015). S-nitrosylation triggers ABI5 degradation to promote seed germination and seedling growth. Nat. Commun. 6, 8669. 10.1038/ncomms9669 26493030 PMC4639896

[B2] Astier J. Kulik A. Koen E. Besson-Bard A. Bourque S. Jeandroz S.. (2012). Protein S-nitrosylation: what's going on in plants? Free Radic. Biol. Med. 53, 1101–1110. 10.1016/j.freeradbiomed.2012.06.032 22750205

[B3] Bai X. Yang L. Tian M. Chen J. Shi J. Yang Y.. (2011). Nitric Oxide enhances desiccation tolerance of recalcitrant antiaris toxicaria seeds via protein S-nitrosylation and carbonylation. PLoS ONE 6:e20714. 10.1371/journal.pone.0020714 21674063 PMC3107241

[B4] Baxter A. Mittler R. Suzuki N. (2014). ROS as key players in plant stress signalling. J. Exp. Bot. 65, 1229–1240. 10.1093/jxb/ert375 24253197

[B5] Begara-Morales J. C. Sánchez-Calvo B. Chaki M. Valderrama R. Mata-Pérez C. López-Jaramillo J.. (2014). Dual regulation of cytosolic ascorbate peroxidase (APX) by tyrosine nitration and S-nitrosylation. J. Exp. Bot. 65, 527–538. 10.1093/jxb/ert396 24288182 PMC3904709

[B6] Beligni M. V. Lamattina L. (2001). Nitric oxide: a non-traditional regulator of plant growth. Trends Plant Sci. 6, 508–509. 10.1016/S1360-1385(01)02156-2 11701377

[B7] Bellin D. Asai S. Delledonne M. Yoshioka H. (2013). Nitric oxide as a mediator for defense responses. Mol. Plant. Microbe. Interact. 26, 271–277. 10.1094/MPMI-09-12-0214-CR 23151172

[B8] Besson-Bard A. Pugin A. Wendehenne D. (2008). New insights into nitric oxide signaling in plants. Annu. Rev. Plant Biol. 59, 21–39. 10.1146/annurev.arplant.59.032607.092830 18031216

[B9] Chaki M. Alvarez de Morales P. Ruiz C. Begara-Morales J. C. Barroso J. B. Corpas F. J.. (2015). Ripening of pepper (*Capsicum annuum*) fruit is characterized by an enhancement of protein tyrosine nitration. Ann. Bot. 116, 637–647. 10.1093/aob/mcv016 25814060 PMC4577987

[B10] Cheng T. Chen J. EF A. Wang P. Wang G. Hu X.. (2015). Quantitative proteomics analysis reveals that S-nitrosoglutathione reductase (GSNOR) and nitric oxide signaling enhance poplar defense against chilling stress. Planta 242, 1361–1390. 10.1007/s00425-015-2374-5 26232921

[B11] Clark D. Durner J. Navarre D. A. Klessig D. F. (2000). Nitric oxide inhibition of tobacco catalase and ascorbate peroxidase. Mol. Plant. Microbe. Interact. 13, 1380–1384. 10.1094/MPMI.2000.13.12.1380 11106031

[B12] Correa-Aragunde N. Foresi N. Delledonne M. Lamattina L. (2013). Auxin induces redox regulation of ascorbate peroxidase 1 activity by S-nitrosylation/denitrosylation balance resulting in changes of root growth pattern in Arabidopsis. J. Exp. Bot. 64, 3339–3349. 10.1093/jxb/ert172 23918967

[B13] Correa-Aragunde N. Foresi N. Lamattina L. (2015). Nitric oxide is an ubiquitous signal for maintaining redox balance in plant cells: regulation of ascorbate peroxidase as a case study. J. Exp. Bot. 66, 2913–2921. 10.1093/jxb/erv073 25750426

[B14] Delledonne M. Zeier J. Marocco A. Lamb C. (2001). Signal interactions between nitric oxide and reactive oxygen intermediates in the plant hypersensitive disease resistance response. Proc. Natl. Acad. Sci. U.S.A. 98, 13454–13459. 10.1073/pnas.231178298 11606758 PMC60892

[B15] de Pinto M. C. Locato V. De Gara L. (2012). Redox regulation in plant programmed cell death. Plant. Cell Environ. 35, 234–244. 10.1111/j.1365-3040.2011.02387.x 21711357

[B16] de Pinto M. C. Locato V. Sgobba A. Romero-Puertas M. D. C. Gadaleta C. Delledonne M.. (2013). S-nitrosylation of ascorbate peroxidase is part of programmed cell death signaling in tobacco bright yellow-2 cells. Plant Physiol. 163, 1766–1775. 10.1104/pp.113.222703 24158396 PMC3846137

[B17] Du S. Zhang R. Zhang P. Liu H. Yan M. Chen N.. (2015). Elevated CO_2_-induced production of nitric oxide (NO) by NO synthase differentially affects nitrate reductase activity in Arabidopsis plants under different nitrate supplies. J. Exp. Bot. 67, 893–904. 10.1093/jxb/erv506 26608644

[B18] Du S. Zhang Y. Lin X. Wang Y. Tang C. (2008). Regulation of nitrate reductase by nitric oxide in Chinese cabbage pakchoi (*Brassica chinensis* L.). Plant. Cell Environ. 31, 195–204. 10.1111/j.1365-3040.2007.01750.x 18028279

[B19] Feechan A. Kwon E. Yuri B. Wang Y. Pallas J. Loake G. (2005). A central role for S-nitrosothiols in plant disease resistance. Proc. Natl. Acad. Sci. U.S.A. 102, 8054–8059. 10.1073/pnas.0501456102 15911759 PMC1142375

[B20] Fröhlich A. Durner J. (2011). The hunt for plant nitric oxide synthase (NOS): is one really needed? Plant Sci. 181, 401–404. 10.1016/j.plantsci.2011.07.014 21889045

[B21] Frungillo L. Skelly M. J. Loake G. J. Spoel S. H. Salgado I. (2014). S-nitrosothiols regulate nitric oxide production and storage in plants through the nitrogen assimilation pathway. Nat. Commun. 5, 1–10. 10.1038/ncomms6401 25384398 PMC4229994

[B22] Gaupels F. Kuruthukulangarakoola G. T. Durner J. (2011). Upstream and downstream signals of nitric oxide in pathogen defence. Curr. Opin. Plant Biol. 14, 707–714. 10.1016/j.pbi.2011.07.005 21816662

[B23] Gibbs D. J. Bacardit J. Bachmair A. Holdsworth M. J. (2014). The eukaryotic N-end rule pathway: conserved mechanisms and diverse functions. Trends Cell Biol. 24, 603–611. 10.1016/j.tcb.2014.05.001 24874449

[B24] Gupta K. J. Fernie A. R. Kaiser W. M. van Dongen J. T. (2011a). On the origins of nitric oxide. Trends Plant Sci. 16, 160–168. 10.1016/j.tplants.2010.11.007 21185769

[B25] Gupta K. J. Hebelstrup K. H. Mur L. A. Igamberdiev A. U. (2011b). Plant hemoglobins: important players at the crossroads between oxygen and nitric oxide. FEBS Lett. 585, 3843–3849. 10.1016/j.febslet.2011.10.036 22036787

[B26] Halliwell B. Gutteridge J. M. C. (2007). Free Radicals in Biology and Medicine. Oxford: Oxford University Press.

[B27] Holzmeister C. Gaupels F. Geerlof A. Sattler M. Durner J. (2015). Differential inhibition of arabidopsis superoxide dismutases by peroxynitrite-mediated tyrosine nitration. J. Exp. Bot. 66, 989–999. 10.1093/jxb/eru458 25428993 PMC4321555

[B28] Jimenez A. Hernandez J. A. Del Rio L. A. Sevilla F. (1997). Evidence for the presence of the ascorbate-glutathione cycle in mitochondria and peroxisomes of pea leaves. Plant Physiol. 114, 275–284. 12223704 10.1104/pp.114.1.275 PMC158303

[B29] Kovacs I. Lindermayr C. (2013). Nitric oxide-based protein modification: formation and site-specificity of protein S-nitrosylation. Front. Plant Sci. 4:137. 10.3389/fpls.2013.00137 23717319 PMC3653056

[B30] Lindermayr C. Saalbach G. Durner J. (2005). Proteomic identification of S -nitrosylated proteins. Plant Physiol. 137, 921–930. 10.1104/pp.104.058719 15734904 PMC1065393

[B31] Liu L. Hausladen A. Zeng M. Que L. Heitman J. Stamler J. S. (2001). A metabolic enzyme for S-nitrosothiol conserved from bacteria to humans. Nature 410, 490–494. 10.1038/35068596 11260719

[B32] Lozano-Juste J. Colom-Moreno R. León J. (2011). *In vivo* protein tyrosine nitration in *Arabidopsis thaliana*. J. Exp. Bot. 62, 3501–3517. 10.1093/jxb/err042 21378116 PMC3130175

[B33] Neill S. Bright J. Desikan R. Hancock J. Harrison J. Wilson I. (2008). Nitric oxide evolution and perception. J. Exp. Bot. 59, 25–35. 10.1093/jxb/erm218 17975211

[B34] Ortega-Galisteo A. P. Rodríguez-Serrano M. Pazmiño D. M. Gupta D. K. Sandalio L. M. Romero-Puertas M. C. (2012). S-nitrosylated proteins in pea (*Pisum sativum* L.) leaf peroxisomes: changes under abiotic stress. J. Exp. Bot. 63, 2089–2103. 10.1093/jxb/err414 22213812 PMC3295397

[B35] Palmieri M. C. Sell S. Huang X. Scherf M. Werner T. Durner J.. (2008). Nitric oxide-responsive genes and promoters in *Arabidopsis thaliana*: a bioinformatics approach. J. Exp. Bot. 59, 177–186. 10.1093/jxb/erm345 18272923

[B36] Perazzolli M. Dominici P. Romero-Puertas M. C. Zago E. Zeier J. Sonoda M.. (2004). Arabidopsis nonsymbiotic hemoglobin AHb1 modulates nitric oxide bioactivity. Plant Cell 16, 2785–2794. 10.1105/tpc.104.025379 15367716 PMC520971

[B37] Perazzolli M. Romero-Puertas M. C. Delledonne M. (2006). Modulation of nitric oxide bioactivity by plant haemoglobins. J. Exp. Bot. 57, 479–488. 10.1093/jxb/erj051 16377734

[B38] Petrov V. D. Van Breusegem F. (2012). Hydrogen peroxide-a central hub for information flow in plant cells. AoB Plants 2012:pls014. 10.1093/aobpla/pls014 22708052 PMC3366437

[B39] Rockel P. Strube F. Rockel A. Wildt J. Kaiser W. M. (2002). Regulation of nitric oxide (NO) production by plant nitrate reductase *in vivo* and *in vitro*. J. Exp. Bot. 53, 103–110. 10.1093/jexbot/53.366.103 11741046

[B40] Romero-Puertas M. C. Corpas F. J. Sandalio L. M. Leterrier M. Rodríguez-Serrano M. Del Río L. A.. (2006). Glutathione reductase from pea leaves: response to abiotic stress and characterization of the peroxisomal isozyme. New Phytol. 170, 43–52. 10.1111/j.1469-8137.2006.01643.x 16539602

[B41] Romero-Puertas M. C. Laxa M. Mattè A. Zaninotto F. Finkemeier I. Jones A. M. E.. (2007). S-nitrosylation of peroxiredoxin II E promotes peroxynitrite-mediated tyrosine nitration. Plant Cell 19, 4120–4130. 10.1105/tpc.107.055061 18165327 PMC2217656

[B42] Romero-Puertas M. C. Rodríguez-Serrano M. Sandalio L. M. (2013). Protein S-nitrosylation in plants under abiotic stress: an overview. Front. Plant Sci. 4:373. 10.3389/fpls.2013.00373 24065977 PMC3778396

[B43] Rustérucci C. Espunya M. C. Díaz M. Chabannes M. Martínez M. C. (2007). S-nitrosoglutathione reductase affords protection against pathogens in Arabidopsis, both locally and systemically. Plant Physiol. 143, 1282–1292. 10.1104/pp.106.091686 17277089 PMC1820916

[B44] Sakamoto A. Ueda M. Morikawa H. (2002). Arabidopsis glutathione-dependent formaldehyde dehydrogenase is an S-nitrosoglutathione reductase. FEBS Lett. 515, 20–24. 10.1016/S0014-5793(02)02414-6 11943187

[B45] Sandalio L. M. Romero-Puertas M. C. (2015). Peroxisomes sense and respond to environmental cues by regulating ROS and RNS signalling networks. Ann. Bot. 116, 475–485. 10.1093/aob/mcv074 26070643 PMC4577995

[B46] Sehrawat A. Abat J. K. Deswal R. (2013). RuBisCO depletion improved proteome coverage of cold responsive S-nitrosylated targets in *Brassica juncea*. Front. Plant Sci. 4:342. 10.3389/fpls.2013.00342 24032038 PMC3759006

[B47] Serrano I. Romero-Puertas M. C. Rodríguez-Serrano M. Sandalio L. M. Olmedilla A. (2012). Peroxynitrite mediates programmed cell death both in papillar cells and in self-incompatible pollen in the olive (*Olea europaea* L.). J. Exp. Bot. 63, 1479–1493. 10.1093/jxb/err392 22140239 PMC3276107

[B48] Tanou G. Job C. Rajjou L. Arc E. Belghazi M. Diamantidis G.. (2009). Proteomics reveals the overlapping roles of hydrogen peroxide and nitric oxide in the acclimation of citrus plants to salinity. Plant J. 60, 795–804. 10.1111/j.1365-313X.2009.04000.x 19682288

[B49] Vandelle E. Delledonne M. (2011). Peroxynitrite formation and function in plants. Plant Sci. 181, 534–539. 10.1016/j.plantsci.2011.05.002 21893249

[B50] Yang H. Mu J. Chen L. Feng J. Hu J. Li L.. (2015). S-Nitrosylation positively regulates ascorbate peroxidase activity during plant stress responses. Plant Physiol. 167, 1604–1615. 10.1104/pp.114.255216 25667317 PMC4378166

[B51] Yun B.-W. Feechan A. Yin M. Saidi N. B. B. Le Bihan T. Yu M.. (2011). S-nitrosylation of NADPH oxidase regulates cell death in plant immunity. Nature 478, 264–268. 10.1038/nature10427 21964330

